# Supplementary material for: Cortical cerebrovascular and metabolic perturbations in the 5xFAD mouse model of Alzheimer’s disease
Source: Front Aging Neurosci. 2023 Jul 18;15:1220036. doi: 10.3389/fnagi.2023.1220036 (PMC10392850; doi:10.3389/fnagi.2023.1220036)
Supplement: Supplementary file 1 [file Data_Sheet_1.docx]

Supplementary Material

Cortical cerebrovascular and metabolic perturbations in the 5xFAD mouse model of Alzheimer’s disease

**Amandine Jullienne^1^, Jenny I. Szu^1^, Ryan Quan^1^, Michelle V. Trinh^1^, Tannoz Norouzi^1^, Brenda P. Noarbe^1^, Amanda A. Bedwell^3^, Kierra Eldridge^3^, Scott C. Persohn^3^, Paul R. Territo^2,3^, and Andre Obenaus^1*^**

*** Correspondence:** Andre Obenaus, PhD: obenausa@uci.edu

## Supplementary Figures


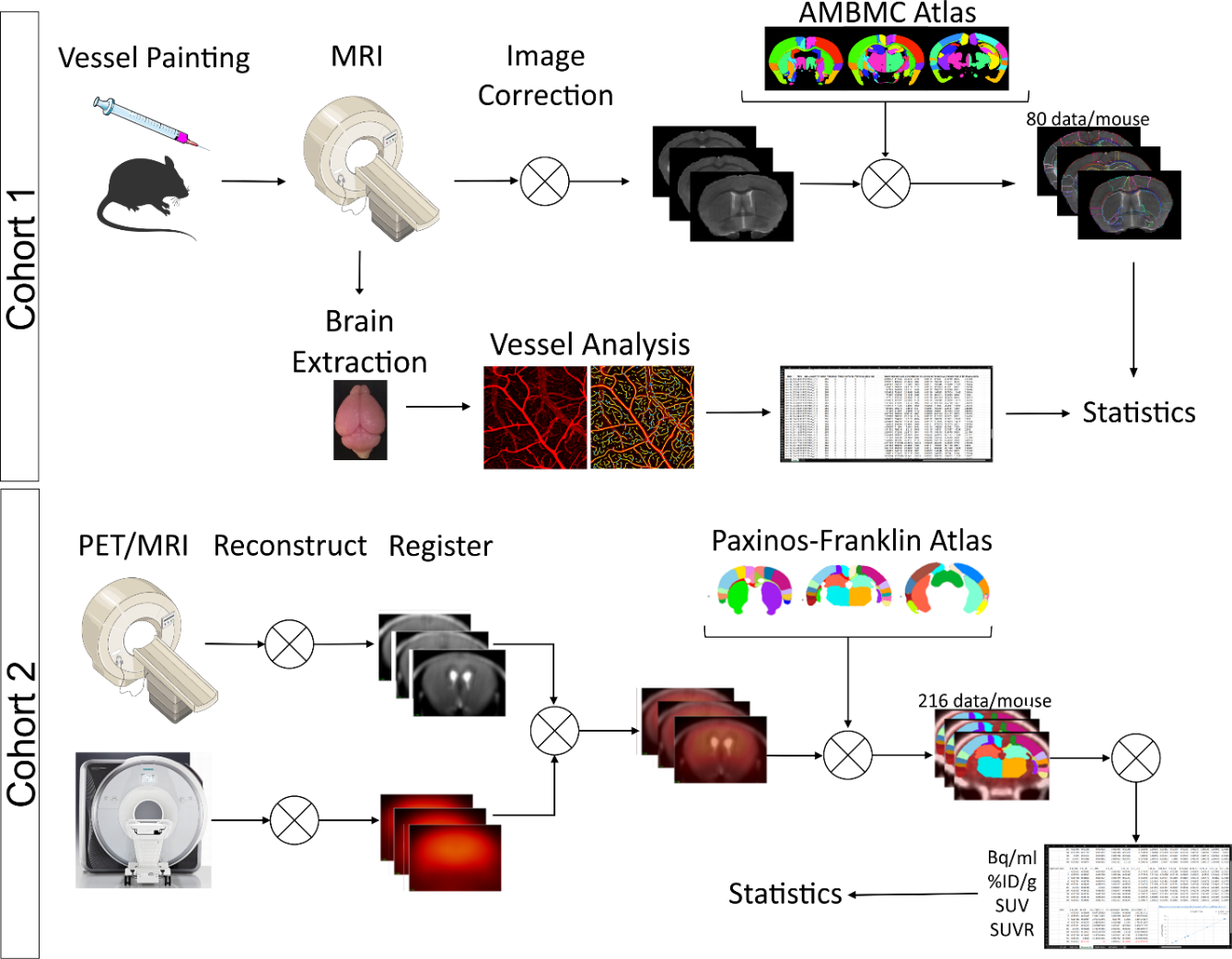


**Supplementary Figure 1:** Experimental design. Two cohorts of age- and sex-matched 5xFAD and WT mice were used for independent experiments. Cohort 1 (4-, 8-, and 12-month-old mice) underwent vessel painting followed by high-resolution ex vivo MRI for vessel network and regional brain volumes analysis. Cohort 2 (4-, 6-, and 12-month-old mice) went through ^18^F-FDG-PET/MRI for regional brain metabolic analyses.


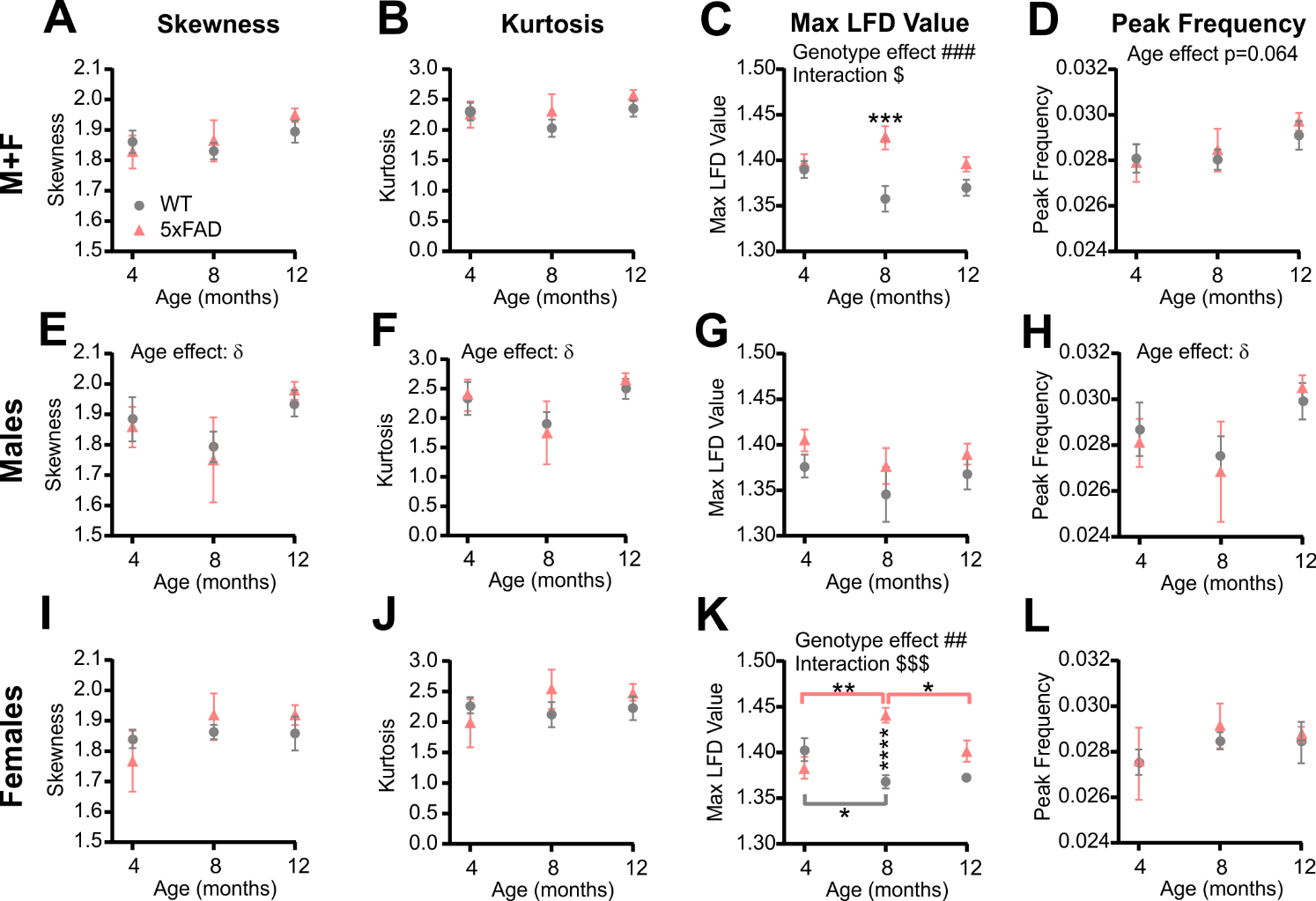


**Supplementary Figure 2:** Metrics associated with the local fractal dimension (LFD) histograms (see Figure 4). Skewness, kurtosis, maximum LFD value, and peak frequency values are shown for males and females combined **(A-D)**, for males only **(E-H)**, and females only **(I-L)**. δ shows a significant effect of age (two-way ANOVA, δ=p<0.05); # shows a significant effect of the genotype (two-way ANOVA, ###=p<0.001); for multiple comparisons across ages and between genotypes (Sidak’s test): *=p<0.05, **=p<0.01, ***=p<0.001, ****=p<0.0001, data shown as mean+SEM.


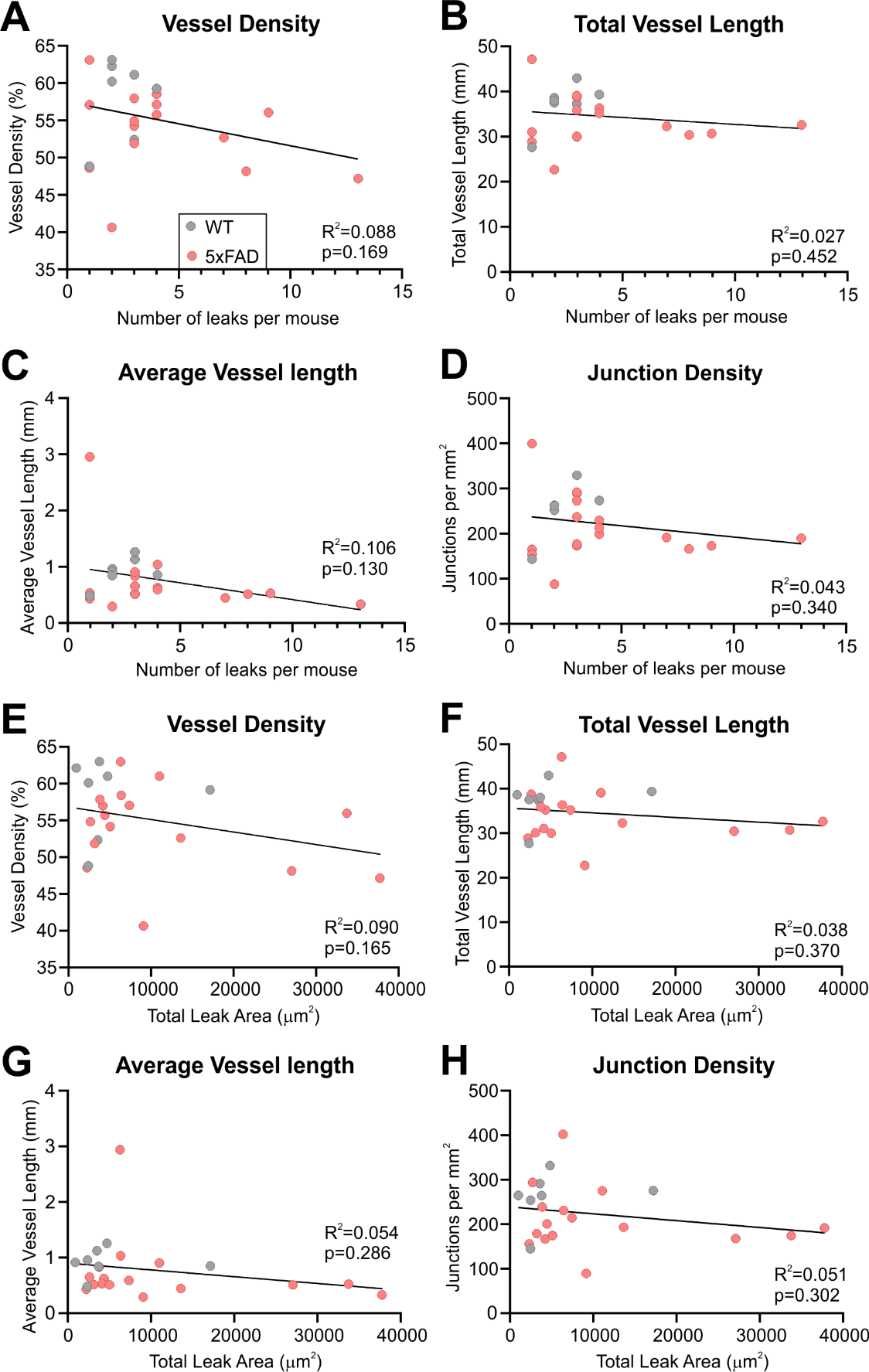


**Supplementary Figure 3:** Correlation plots between classical vessel metrics and number of DiI leaks per mouse (**A-D**) or total leak area per mouse (**E-G**). No correlations were significant. All data were assessed using linear regression modeling and goodness of fit function in the GraphPad software (version 9.5.1).


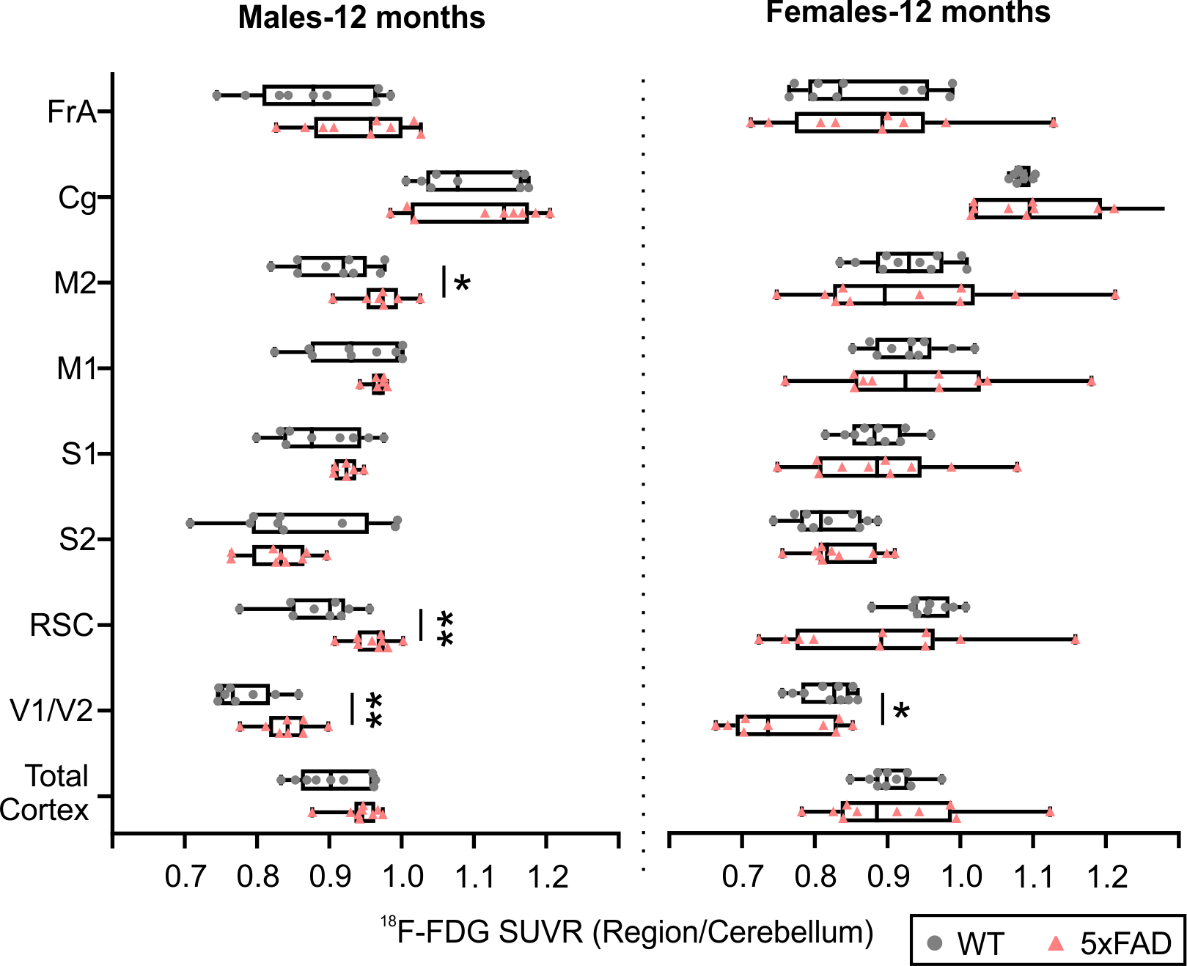


**Supplementary Figure 4:** ^18^F-FDG PET cortical metabolism. In 5xFAD male mice at 12 months of age, the M2, RSC and V1/V2 cortical areas had significantly increased 18F-FDG uptake in contrast to female mice which exhibited decreased uptake, suggesting sex differences. T-tests compared WT and 5xFAD mice with *=p<0.05, **=p<0.01. FrA: frontal association, Cg: cingulate, M1/M2: primary/secondary motor area, S1/S2: primary/secondary sensorimotor area, RSC: retrosplenial dysgranular cortex, V1/V2: primary/secondary visual area.
